# Immunomodulation of *Glycyrrhiza* Polysaccharides In Vivo Based on Microbiome and Metabolomics Approaches

**DOI:** 10.3390/foods14050874

**Published:** 2025-03-04

**Authors:** Yixuan Wu, Jie Sun, Wenjie Xie, Simin Xue, Xinli Li, Jianming Guo, Jinjun Shan, Guoping Peng, Yunfeng Zheng

**Affiliations:** 1 Department of Pharmacy, Nanjing University of Chinese Medicine, Nanjing 210046, China; wyx09272021@163.com (Y.W.); sunjiexueyong@163.com (J.S.); xwenj0107@163.com (W.X.); xuesimin0912@163.com (S.X.); lixinli2023@163.com (X.L.); 320787@njucm.edu.cn (G.P.); 2 National Key Laboratory on Technologies for Chinese Medicine Pharmaceutical Process Control and Intelligent Manufacture, Nanjing 211100, China; 3 Jiangsu Province Engineering Research Center of Classical Prescription, Nanjing University of Chinese Medicine, Nanjing 210023, China; 4 Jiangsu Collaborative Innovation Center of Chinese Medicinal Resources Industrialization, Nanjing University of Chinese Medicine, Nanjing 210023, China; njuguo@njucm.edu.cn; 5 Jiangsu Key Laboratory for High Technology Research of TCM Formulae, Nanjing University of Chinese Medicine, Nanjing 210023, China; 6 Jiangsu Key Laboratory of Pediatric Respiratory Disease, Institute of Pediatrics, Nanjing University of Chinese Medicine, Nanjing 210023, China; jshan@njucm.edu.cn

**Keywords:** *Glycyrrhiza* polysaccharides, immunocompromised rats, untargeted metabolomics, SCFAs, gut microbiota

## Abstract

*Glycyrrhiza uralensis Fisch.* is a medicinal herb that can be added to food to provide therapeutic effects and reduce the burden of medications. Herein, the immunomodulatory effects of *Glycyrrhiza* polysaccharides (GPs) were verified and illustrated by intervening immunocompromised rats treated with different doses of GPs, which were reflected for adjusting the composition and structure of the intestinal microbiota and altering the metabolic profile. The immunomodulatory effects of GPs were exerted by regulating the intestinal microenvironment. In particular, GPs could promote the growth of probiotic bacteria *Allobaculum*, *norank__o_Clostridia_UCG-014*, *Dubosiella*, and *g__norank_o___RF39* and curb the growth of harmful bacteria *Enterococcus*. The results showed that GPs had a prebiotic effect, which contributed to improving the intestinal environment and maintaining intestinal health. In addition, the content of beneficial differential metabolites was up-regulated, especially short-chain fatty acids, with alanine, aspartate, and glutamate metabolism; arginine biosynthesis; glyoxylate and dicarboxylate metabolism being the most enriched pathways. These metabolic pathways imply the metabolic process of GPs, and the metabolic pathways and differential effector metabolites of it are focused. Overall, the purpose of this article lies in providing support for the application of GPs for regulating immune function.

## 1. Introduction

Owing to its medicinal and edible homologous properties, *Glycyrrhiza* is extensively used as a food additive. For example, *Glycyrrhiza* flavonoids have drawn attention to their promising applications as food preservatives [1]. In addition, glycyrrhizin, a prominent component of *Glycyrrhiza*, can be used as an effective natural sweetener [2]. Furthermore, *Glycyrrhiza* polysaccharides (GPs), identified as one of the active constituents of *Glycyrrhiza*, warrant further investigation for their health benefits.

GPs are macromolecular compounds derived from the rhizome of *Glycyrrhiza*. The biological activity of GPs is influenced by their structure, and several key factors are molecular weight, monosaccharide composition, and glycosidic bond configuration [3]. Notably, polysaccharides comprising a higher proportion of acidic components exhibit better activity [4]. The structural and functional properties of GPs are also influenced by the extraction method employed [5]. The immunomodulatory effects of GPs have emerged as a substantial area of research interest. These polysaccharides have demonstrated the ability to promote macrophage proliferation and phagocytosis [6], modulate cytokine secretion [7], enhance overall immune system function [8], and bolster the resistance of the body to pathogens [9]. The immunomodulatory activity of GPs provides insight into their anti-tumor mechanisms [10]. Furthermore, GPs demonstrate antioxidant activity, which is vital for protecting and enhancing the immune system [11].

However, the complex structure and high molecular weight of GPs pose challenges to their absorption and utilization in the body [12]. Research indicates that polysaccharides, including those derived from Ganoderma lucidum and plantain, resist degradation by human digestive enzymes. Consequently, these polysaccharides reach the large intestine intact and get metabolized by the gut microbiota. A substantial portion of the genetic material of the gut microbiome is dedicated to the assimilation and breakdown of carbohydrates, thereby facilitating the body’s absorption and utilization of polysaccharides [13]. Through microbial fermentation in the intestine, polysaccharides aid in the regulation of carbohydrate metabolism and promote human health [14]. Research has demonstrated that GPs enhance diabetic conditions and immune function by modulating the gut microbiome [15]. These polysaccharides act as a crucial energy source for gut microbiota, contributing to the growth of probiotics and maintaining the integrity of the intestinal barrier [16]. During this process, the gut microbiota engages in intricate metabolic activities, producing a variety of metabolites, including short-chain fatty acids (SCFAs), indole derivatives, and secondary bile acids [17], which are intricately linked to immune function. Studies have shown that Glycyrrhiza polysaccharides can be used as feed to increase the content of short-chain fatty acids in broilers, thereby improving intestinal health and immune function [18,19].

Despite these findings, the interaction between GPs and the intestinal flora remains to be explored. Because of the particularity of the structure, the interaction between GPs and intestinal flora is a research hotspot. Since short-chain fatty acids are important metabolites of the gut microbiota, correspondingly, there are many studies on the polysaccharides-gut microbiota-SCFAs axis. However, the combination of targeted and non-targeted metabolomics to study the changes in metabolites produced by GPs in serum and feces is rarely mentioned. Employing non-targeted and targeted metabolomics can more effectively elucidate the pathways and metabolic signatures involved in the microbial breakdown and utilization of polysaccharides. Herein, we conducted a preliminary characterization of the composition and structure of GPs using spectroscopy, chromatography, and microscopy. Subsequently, 16S rRNA sequencing technology was applied to analyze the intestinal microbiota of immunocompromised rats. This analysis focused on determining the composition and structure of the microbiota, especially on identifying genera influenced by GPs. Metabolite profiling was then conducted to identify differential metabolites, thereby elucidating the mechanisms through which GPs modulate immune function. In conclusion, our findings demonstrate that GPs enhance immune function by rectifying imbalances in the intestinal microbiota and addressing metabolic disorders. The findings of this study will act as a benchmark for creating GPs as functional foods and nutraceuticals.

## 2. Materials and Methods

### 2.1. Reagents and Materials

The *Glycyrrhiza* samples obtained from Hangjin Banner, Ordos City, Inner Mongolia, were identified by Professor Hui Yan as the roots and rhizomes of *G. uralensis* Fisch. Reference materials with purities exceeding 98%, including rhamnose, galacturonic acid, glucose, galactose, and arabinose, were sourced from Shanghai Yuanye Bio-Technology Co., Ltd., based in Shanghai, China. Lentinan and cyclophosphamide were purchased from the same places above. SCFA standards such as acetate, propionate, butyrate, isobutyrate, 2-methyl-butyrate, and isovalerate were acquired from Aladdin Co., Ltd. (Shanghai, China). Formic acid with HPLC grade was obtained from ACS (Washington, DC, USA), and Merck (Darmstadt, Germany) provided acetonitrile and methanol of HPLC and GC–MS grades.

### 2.2. Preparation of Glycyrrhiza Polysaccharides

The extraction and alcohol precipitation methods of GPs were the results of experiment. Five kilograms of *Glycyrrhiza* samples were extracted thrice using deionized water at a ratio of 1:10 for 2 h. The resulting filtrate was sequentially adsorbed by polyamide and microporous adsorption resin, and the effluent was gathered and concentrated. Absolute ethanol was added to the concentrate until the alcohol content was 65%, and then it was centrifuged at 10,000 rpm for 10 min to eliminate the supernatant. The sample solution was treated with Sevag reagent to remove proteins until no visible protein precipitate remained in the middle layer [20]. The process of freeze-drying produced 76.2 g of GPs, with a yield of 1.52%.

### 2.3. Measurement of Molecular Weight

Gel permeation chromatography was employed to determine the molecular weight of the polysaccharides, following a previously reported method with some minor changes [21]. For the determination of polysaccharide molecular weight, a high-performance liquid chromatography system (Waters e2695) with a TSK-G3000 PWXL column (7.8 mm × 30 cm, 6 μm; TOSOH Bioscience, Tokyo, Japan) was used. After dissolving GPs in pure water, it was filtered through 0.45 μm membranes. The sample was subsequently injected, with ultrapure water serving as the mobile phase, and the flow was set to 0.6 mL/min at 30 °C. The polysaccharide peak was identified using an ELSD, and the temperature of the drift tube was maintained at 110 °C. Dextrans with specified molecular weights (1000, 5000, 11,600, 23,800, 80,900, 148,000, and 273,000 Da; Aladdin, Shanghai, China) were utilized to create the calibration curve under identical conditions. The regression equation was plotted with the peak time on the abscissa and the molecular weight of the dextran standard expressed in logarithmic form as the ordinate. The retention time of the chromatographic peaks is factored into the regression equation to calculate the molecular weight of GPs.

### 2.4. Monosaccharide Composition Analysis

The monosaccharide composition of GPs was analyzed using a high-performance liquid chromatography system (Waters e2695) with a Hedra ODS-2 column (4.6 × 250 mm, 5 μm; Hanbon Sci. & Tech., Huaiyin, China). The sample was dissolved in water, hydrolyzed with 2 M trifluoroacetic acid (TFA) at 100 °C for 6 h, dried with nitrogen, and 1 mL of methanol was added to remove excess TFA, and this step was repeated three times. Afterward, the sample was then dissolved again in 1 mL of ultra-pure water and derivatized with 10% PMP methanol solution at 70 °C for 45 min. The PMP was then extracted with chloroform and removed, and the uppermost aqueous layer was collected and passed through a 0.45 μm syringe filter. The volume for injection was established at 10 μL. A gradient method with acetonitrile (solvent A) and ammonium acetate (solvent B) served as the mobile phase, with the column temperature at 30 °C and detection wavelength at 254 nm, operating at a flow rate of 1.0 mL/min. The following settings were used for the elution gradient: from 0 to 65 min, 15–25% A. Rhamnose, galacturonic acid, glucose, galactose, and arabinose were employed as standardized compounds for quantitative measurement.

### 2.5. Fourier Transform Infrared Spectroscopy Analysis

After the GPs were ground into a fine powder, it is mixed with KBr particles (spectral grade) and pressed into thin sheets by a tablet press. The absorbance of GPs was measured using a Fourier transform infrared spectrometer (Nicolet IR100, Thermo Scientific, San Jose, CA, USA) spanning 4000–400 cm^−1^ [22].

### 2.6. Scanning Electron Microscopy Analysis

Freeze-dried GPs were positioned on a metal carrier stage and analyzed using a scanning electron microscope model S4800 (Hitachi High-Tech Corporation, Tokyo, Japan), capturing images at various magnifications with a voltage of 10.0 kV.

### 2.7. Animal Experiment

We acquired 42 male Wistar rats that were free of specific pathogens, weighed between 160 and 180 g, and were aged 6–8 weeks from Shanghai Slack Laboratory Animal Co., Ltd., Shanghai, China, (SCXK [hu] 2022–0004). The rats were housed in the Laboratory Animal Center of Nanjing University of Chinese Medicine, and all experiments were approved by the Animal Ethics Committee of the Nanjing University of Chinese Medicine (Ethics No. 202404A053). After a week of adaptation through feeding, 42 rats were randomly assigned to five groups, with no fewer than 8 rats in each group, namely, the control group (labeled as CON), the model group (labeled as MOD), the lentinan group (labeled as POS), the low-dose polysaccharide group (labeled as GP-L) and the high-dose polysaccharide group (labeled as GP-H). During the two-week treatment period, rats in the control and model groups received sterile water orally, whereas those in the GP-L, GP-H, and POS groups were administered low-dose polysaccharide (0.52 g/kg/d), high-dose polysaccharide (2.10 g/kg/d) and lentinan (40 mg/kg/d), respectively. For the initial 3 days, rats in the MOD, GP-L, GP-H, and POS groups received intraperitoneal injections of cyclophosphamide (60 mg/kg/d) to create an immunocompromised rat model. The control group was given an equivalent volume of sterile saline. The dosages for the GP-L, GP-H, and POS groups were established using our prior experimental findings and references [23]. At the conclusion of the experiment, all rats were euthanized to gather their blood, spleens, thymus, intestinal tissues, and fecal samples. The spleens and intestinal tissues were split into two sections: one was preserved in 4% paraformaldehyde and the other was kept at −80 °C for subsequent analysis. Visceral indexes (mg/g) were calculated as [Organ weight (mg)/body weight (g)].

### 2.8. Histological and Immunofluorescence Analysis

The ileum and spleen were preserved in 4% paraformaldehyde for 24 h, then embedded in paraffin, sectioned, and dehydrated using a series of graded ethanol solutions [24]. The morphological structures of the ileum and spleen paraffin sections, stained with hematoxylin and eosin (H&E), were assessed using a Pannoramic MIDI system equipped with a brightfield digital slide scanner. K-Viewer KFSlideOS 1.0.5 software was utilized for the histological analysis.

The intestinal tissues embedded in paraffin were deparaffinized, re-hydrated, and rinsed with distilled water. Antigen retrieval was performed, followed by blocking with serum. Then, primary antibodies ZO-1 and occludin were applied and incubated at 4 °C [25]. The incubation for the mixture was 50 min after secondary antibodies were added. The nuclei underwent re-stained with DAPI and enclosed, and images were obtained using a fluorescence microscope (Olympus, Tokyo, Japan). The expression of the antibodies was determined using Image J software (1.53 q).

### 2.9. Measurement of Inflammatory Cytokines

Serum levels of IFN-γ, IgG, IL-2, IL-6, and IL-10 were quantified using ELISA kits from Elabscience Biotechnology Co., Ltd., Nanjing, China., following the provided guidelines.

### 2.10. Faecal Sample Preparation and 16S rRNA Sequencing

After the last dose, fecal samples were gathered and stored at −80 °C in a refrigerator. These samples were then sent to Majorbio Inc. in Shanghai, China, for the analysis of the gut microbiota composition using 16S rRNA sequencing on an Illumina MiSeq platform. The paired-end reads from Illumina sequencing were first merged based on their overlap, and then, the sequences were subjected to quality regulation and filtering. Operational taxonomic unit aggregation and species classification were conducted following sample differentiation. The microbiological results were analyzed at http://www.majorbio.com (accessed on 1 August 2024).

### 2.11. Preparation of Serum Samples for Metabolomics Analysis

First, 200 μL of methanol on ice with 12.5 μg/mL myristic acid-1,2-^13^C2 was added to 50 μL of serum. The mixture underwent vortexing for 3 min and was centrifuged at 18,000 rpm for 10 min at 4 °C. Afterwards, 100 μL of the supernatant was relocated to a new tube, where it was evaporated, concentrated, and dried in a centrifuge concentrator. Afterward, 30 μL of pyridine, which contained 10 mg/mL methoxyamine hydrochloride, was introduced, then mixed using a vortex for 5 min and shaken at 300 rpm for 1.5 h at 30 °C. Following this, 30 μL of BSTFA was added and mixed at 300 rpm for 0.5 h more at 37 °C [26].

After the derivatization process, the mixture was centrifuged at 18,000 rpm for 10 min before being injected into a GC–MS (Trace 1310-TSQ 8000 Evo, Thermo Fisher, San Jose, CA, USA) using conditions from a previously reported method [27]. Derivatization processes must take place in a dry setting. According to the protocol mentioned above, serum quality control samples were prepared and derivatized, and they were processed after every 10 samples to observe the peak time and elution sequence of the metabolites.

### 2.12. Untargeted Serum Metabolomics

Raw GC-MS data files were transformed into ABF format and uploaded into the MS-DIAL v4.80 program for detection of peaks, recognition, and contrast with the Fiehn Lab database. MetaboAnalyst 6.0 was utilized to standardize and analyze the data (http://www.metaboanalyst.ca accessed on 18 July 2024). MetaboAnalyst was used to perform the statistical significance tests for identical metabolite screening, metabolic pathway analysis, and cluster analysis. Metabolites with a *p*-value < 0.05, FC > 1.5, FC < 0.67, and VIP > 1 were considered potential differential metabolites.

### 2.13. Targeted Serum and Faecal Metabolomics

After adding 70 μL of acetonitrile to 30 μL of serum, the mixture was vortexed for 3 min and centrifuged at 13,000 rpm for 10 min. A sample of approximately 10 mg of feces was combined with 1.0 mL of 70% acetonitrile, homogenized for 5 min, and centrifuged at 13,000 rpm for 10 min.

The supernatant was added to a 1.5 mL centrifuge tube along with 40 μL of supernatant, 5 μL of d3-hexanoic acid at 10 μg/mL, 20 μL of 200 mM 3 NPH, and 20 μL of 120 mM EDC·HCl-6% pyridine solution. The mixture was subjected to vortexing for 3 min, reacted for 30 min at 40 °C, and centrifuged for 10 min at 18,000 rpm, and an injection of the supernatant was prepared for analysis.

Standard solution preparation involved accurate weighing of acetate, propionate, butyrate, isobutyrate, 2-methylbutyrate, and isovalerate into a 5-mL volumetric flask, with methanol added for subsequent use. Then, 10 μL of SCFAs were mixed, respectively, and methanol was half-diluted to obtain the standard solution of each gradient concentration, and 40 μL of the mixed standard solution of each concentration was taken and processed with the sample at the same step.

### 2.14. Statistical Analysis

GraphPad Prism (9.5.1) and SPSS (Version 22) software were used for all statistical analyses, with results shown as the mean ± standard deviation. The Student’s *t*-test, one-way ANOVA, and Shapiro–Wilk test were utilized to examine the statistical differences between groups. Differences were considered significant if the *p*-value was below 0.05.

## 3. Results and Discussion

### 3.1. Characterisation of Glycyrrhiza Polysaccharide

#### 3.1.1. Chemical Characterisation of GPs

**Figure 1:**
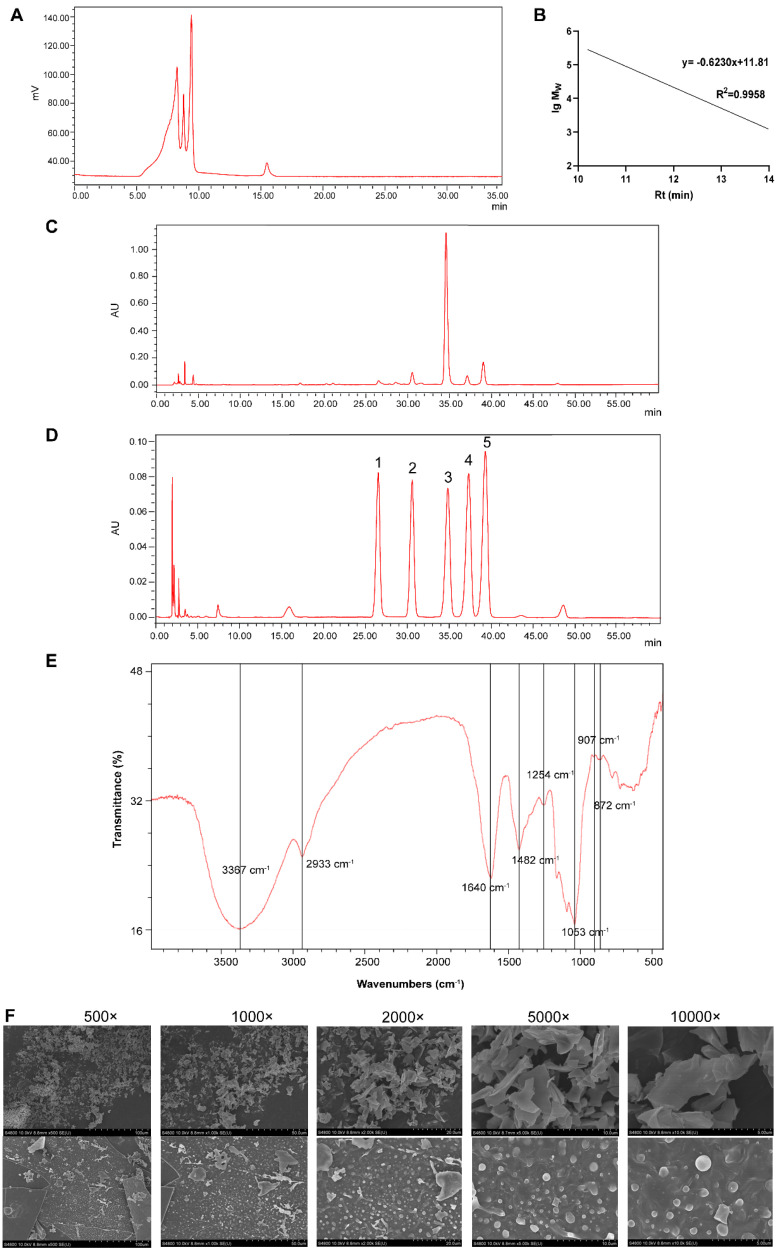
Chemical characterization of GPs. (**A**) High-performance gel permeation chromatography profiles of GPs on a TSK gel PW_XL_ 3000 column. (**B**) Molecular weight (lg M_W_) vs. retention time (Rt) curves. (**C**,**D**) Monosaccharide profiles of standards and GPs by HPLC-PDA: (1) rhamnose, (2) galacturonic acid, (3) glucose, (4) galactose, and (5) arabinose. (**E**) FT-IR spectrum. (**F**) SEM images of GPs at different magnifications.

The total sugar, protein, and uronic acid content of GPs was 77.05% (Figure 1A), 1.56% (Figure S1B), and 13.34% (Figure S1C), respectively. The retention time was brought into the dextran molecular weight standard curve (Figure 1A,B), and the molecular weight of GPs was calculated (Table 1). GPs comprised rhamnose, galacturonic acid, glucose, galactose, and arabinose with molar masses of 1.00, 2.80, 31.8, 1.67, and 3.73, respectively (Figure 1C,D).

#### 3.1.2. FT-IR Analysis of GPs

The FT-IR spectra of GPs are shown in Figure 1E. It was determined that the stretching vibration of −OH was responsible for the strong and wide absorption peak at 3367 cm^−1^, indicating the presence of intermolecular hydrogen bonds. The absorption peak at 2933 cm^−1^ was owing to the stretching vibration of C−H. Moreover, the stretching vibrations of C=O, C−O, and O−H caused 1640, 1428, and 1254 cm^−1^ peaks, respectively, indicating that GPs contained uronic acid. The absorption peaks at 1053, 907, and 872 cm^−1^ were associated with β-type glycosidic bonds and the pyranose ring. Valuable insights into the structural makeup and bonding configurations of GPs were revealed by these spectroscopic features.

#### 3.1.3. Surface Morphological Observation

The SEM results shown in Figure 1F indicate that the spatial structure of GPs was mainly sheet-like, with scattered flakes and different sizes. The surface level was different, and spherical particles could be seen, which may be directly related to the monosaccharide composition, ligation mode, and high-order spatial structure of polysaccharides.

### 3.2. GPs Improve the Organ Index in Immunocompromised Rats

**Figure 2:**
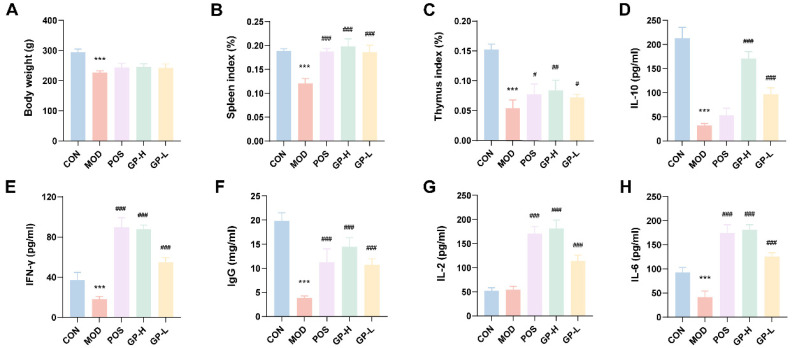
GPs improved the organ index, immune index, and inflammatory cytokine secretion in immunocompromised rats. (**A**) Body weight, (**B**) spleen index, (**C**) thymus index, (**D**) IL-10, (**E**) IFN-γ, (**F**) IgG, (**G**) IL-2, and (**H**) IL-6. Data were expressed as mean ± standard deviation (*n* = 6). *** *p* < 0.001 vs. the control group; ^#^ *p* < 0.05, ^##^ *p*< 0.01, ^###^ *p* < 0.001 vs. the model group.

The body weights of the rats in the MOD group were markedly lower (Figure 2) than those of the CON group, and after the intervention using lentinan, low-dose polysaccharide, and high-dose polysaccharide, the body weights were increased compared with those of the MOD group. Consistently, the spleen index in the MOD group was considerably reduced compared with that in the CON group (*p* < 0.001, Figure 2B), confirming that successful implementation of the immunosuppression model was achieved. The spleen indices of all treated groups were considerably improved compared with those of the MOD group, in which the spleen index of the GPs groups was even higher than that of the CON group, which indicated the immunity-modulating effect of GPs. Moreover, the thymus index is essential for evaluating immune function. In comparison to the CON group, the thymus index in the MOD group was significantly decreased (*p* < 0.001, Figure 2C), and compared with the MOD group, the thymus index of the administered groups was considerably changed (*p* < 0.001), of which the high-dose polysaccharides had a more pronounced effect, yet none reached the level of the CON group. The possible reason was that the thymus was more challenging to recover after being damaged.

### 3.3. GPs Affect Cytokine Secretion in Immunocompromised Rats

To determine cytokine expression in serum, we used ELISA kits (Figure 2D–H). IFN-γ is a key marker of inflammation and immune system involvement. IL-6 is an essential pro-inflammatory cytokine that affects the immune system. IL-2 is required for Treg cell differentiation, immunosuppressive function, homeostasis, and survival. IgG is an essential material basis for immune responses. IL-10 is recognized as an inflammatory and immunosuppressive factor. The MOD group showed a marked reduction in the expression levels of IFN-γ, IL-6, IgG, and IL-10 compared to the CON group (*p* < 0.001). However, the serum cytokine levels in the GP-L and GP-H groups were significantly elevated (*p* < 0.05). This suggests that GPs can enhance immune function in rats with compromised immunity.

### 3.4. GPs Alleviate the Spleen and Intestinal Damage

**Figure 3:**
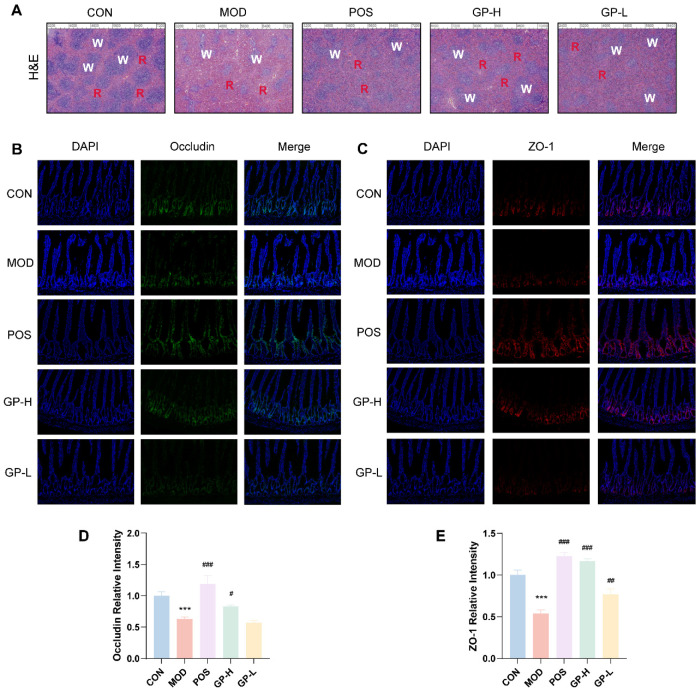
GPs helped to lessen spleen and intestinal damage in rats with impaired immune systems. (**A**) The spleen underwent H&E staining. (**B**) Immunofluorescence of occludin in the intestinal issues. (**C**) Immunofluorescence of ZO-1 in the intestinal issues. (**D**) Statistical graph of occludin. (**E**) Statistical graph of ZO-1. Data are expressed as mean ± standard deviation (*n* = 3). *** *p* < 0.001 vs. the control group; ^#^ *p* < 0.05, ^##^ *p* < 0.01, ^###^ *p* < 0.001 vs. the model group.

In CON rats, the spleen tissue maintained its integrity, with clear boundaries separating the red and white pulps (Figure 3A). However, in MOD rats, the spleen tissue exhibited blurred boundaries between the red and white pulps, and the lymph nodes within the white pulp were not tightly arranged. After the administration of lentinan, low-dose polysaccharide, and high-dose polysaccharide, the spleen tissue structure in rats progressively normalized, with the distinction between red and white pulps becoming clearer.

Using immunofluorescence to detect the expression levels of tight junction proteins occludin and ZO-1, we evaluated the effect of GPs on maintaining the integrity of the intestinal barrier (Figure 3). Results showed that the expression levels of the two proteins in the MOD group were significantly decreased (*p* < 0.001); however, the expression of the two proteins was significantly up-regulated after GP treatment (*p* < 0.05). These results showed that GPs could alleviate the intestinal barrier damage caused by cyclophosphamide, and the effect of a high dose was stronger. Occludin is the first transmembrane protein to form tight junctions in its entirety [28]. Although ZO-1 is not a key protein required for the intestinal barrier, it plays a vital role in repairing it [29]. The C-terminal domain of occludin engages with several intracellular proteins at tight junctions, such as ZO-1, which is essential for incorporating occludin into these junctions [30]. A flaw in the intestinal epithelial tight junction barrier is a crucial pathogenic element that leads to intestinal inflammation, with occludin being vital for maintaining this barrier. Notably, occludin deficiency would increase the selective permeability of macromolecules [31], which would cause damage to the intestinal barrier function.

### 3.5. GPs Regulate the Composition of Intestinal Flora in Immunocompromised Rats

**Figure 4:**
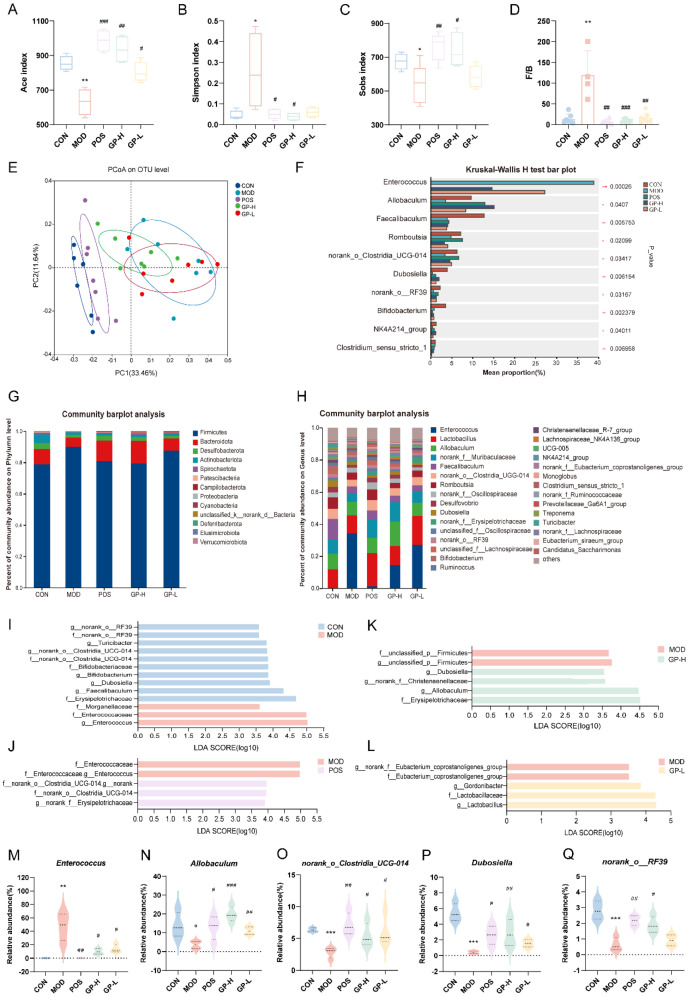
GPs regulated the gut microbial diversity in immunocompromised rats. (**A**) Ace index. (**B**) Simpson index. (**C**) Sobs index (**D**) Firmicutes/Bacteroidetes ratio. (**E**) PCA analysis. (**F**) Multi-group test of variance. (**G**) Phylum-level community barplot analysis. (**H**) Genus-level community barplot analysis. (**I**–**L**) LEfSe analysis between two different groups. (**M**–**Q**) Relative abundance of *Enterococcus*, *Allobaculum*, *norank_o__Clostridia_UCG-014*, *Dubosiella,* and *norank_o_RF39* in different groups. Data are displayed as mean ± standard deviation (*n* ≥ 3). * *p* < 0.05, ** *p* < 0.01, *** *p* < 0.001 vs. the control group; ^#^ *p* < 0.05, ^##^ *p* < 0.01, ^###^ *p* < 0.001 vs. the model group.

In the data analysis, first, alpha diversity reflects the richness and diversity of microbial communities, which can be used to explore the changes in microbial community diversity before and after disease treatment. In Figure 4A–C, the richness and diversity in the MOD group were found to be notably lower than those in the CON group, as shown by alpha-diversity analysis, each administration group showed a marked improvement in richness and diversity compared to the MOD group, and it was even superior to the CON group. Beta-diversity can analyze the similarities or differences of different treatment groups in the overall community structure. The samples in the CON group were aggregated, with little difference and high similarity. In addition, in Figure 4E, the samples from the groups were distant from one another, and a notable difference was observed between the CON and MOD groups. Moreover, the POS and CON groups were extremely close to each other, indicating that the overall structure of the community was similar. Notably, the GP administration groups had a tendency to be far away from the MOD group and close to the CON group, suggesting that GPs can regulate the composition of the intestinal flora in immunocompromised rats.

Analysis of species composition revealed that, at the phylum level (Figure 4G), the flora of all experimental groups mainly comprised the phyla Firmicutes and Bacteroidota. The ratio of Firmicutes to Bacteroidota (*F/B*) was higher in the MOD group than in the CON group (Figure 4D), indicating that the ecological environment of the flora was dysfunctional. However, the *F/B* was regressed after intervention. Previous studies have shown that when *Firmicutes* abundance, *Bacteroidota* abundance decreases and the ratio of these two increases, it might lead to obesity, irritable bowel syndrome, and rheumatoid arthritis, etc. [32]. At the genus level, GPs facilitated the increase of beneficial bacteria *Lactobacillus* [33], *Allobaculum* [34], *Dubosiella*, and *Ruminococcus* and reduced the growth of *Enterococcus* [35] (Figure 4H). Species difference analysis showed that *Enterococcus*, *Allobaculum*, *norank_o__Clostridia_UCG-014*, *Dubosiella,* and *norank_o__RF39* were the five genera with higher abundance than the genera that differed between groups (Figure 4F). After the intervention of GPs, the proportion of *Enterococcus* in the intestinal tract of immunocompromised rats decreased, and the proportion of the other four probiotics significantly increased (*p* < 0.05, Figure 4M–Q).

LEfSe linear discriminant analysis was applied to find species that significantly affected group classification (Figure 4I–L). The LDA value quantified the impact of species on the varying effects. At an LDA score of 3.5, there were 10 taxa impacted in the CON group as opposed to the MOD group, including *g__norank_o___RF39*, *g__Turicibacter*, *g__norank__o___Clostridia_UC -G-014*, *g__Bifidobacterium*, *g__Dubosiella* and *g__Facalibaculum*. Relative to the MOD group, four taxa were altered in the GP-H group, including *g__Dubosiella*, *g__ Allobaculum*. Relative to the MOD group, three taxa were altered in the GP-L group, including *g__Gordonibacter* and *g__Lactobacillus*.

*Enterococcus* may be potentially virulent and drug-resistant, which allows them to function as serious pathogens [36]. *Allobaculum* attenuates inflammatory responses, oxidative stress, and apoptosis [37]. The modulation of *Allobaculum* and associated SCFAs by ginseng-neutral polysaccharides may contribute to their anti-tumor effects [34]. *norank_o__Clostridia_UCG-014* is involved in the relief of colitis symptoms and contributes to the protection of the intestinal barrier [33,38]. The abundance of *norank__o_Clostridia_UCG-014* is reduced in the intestinal flora of rats with rheumatoid arthritis and elevated with the recovery of immune function in immunocompromised mice [39,40]. *Dubosiella newyorkensis* can modulate immune tolerance in colitis and may be a key marker colony of polysaccharides for obesity alleviation [41,42]. These findings indicate that our results align with those from earlier research. In conclusion, the structure and composition of the intestinal flora of the immunocompromised rats changed with the intervention of GPs. Subsequently, probiotic bacteria abundance increased, and harmful bacteria abundance decreased. The intestinal tract serves as the largest immune organ in the body. Consequently, GPs modulate immune responses by adjusting the structure and makeup of gut microbiota.

### 3.6. Serum Metabolic Profiling and Metabolic Pathway Analysis

#### 3.6.1. Serum Metabolic Profiling

**Figure 5:**
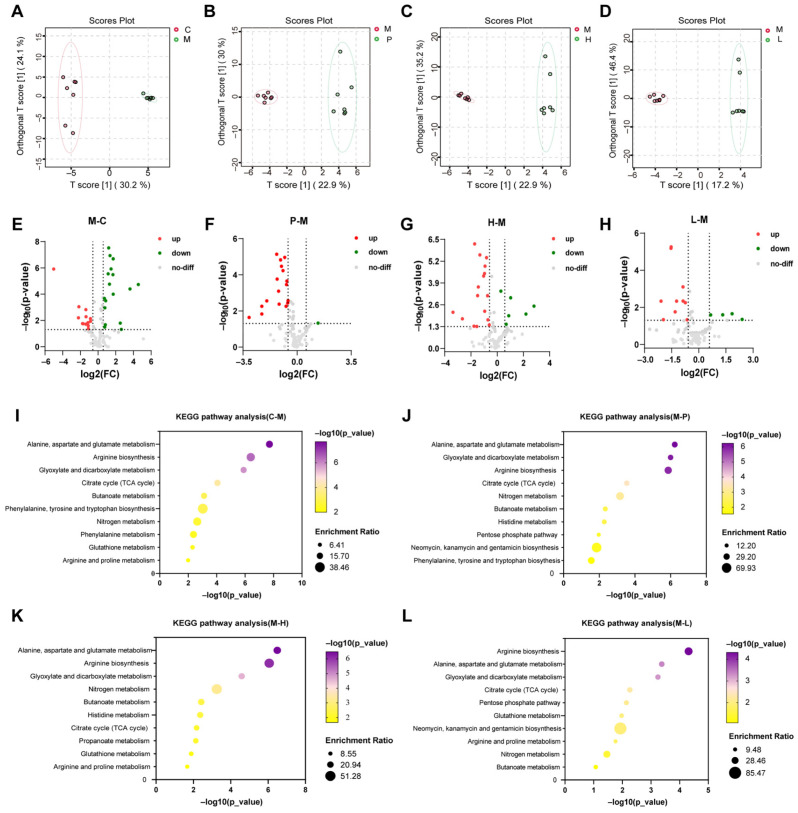
Untargeted metabolomics analysis of serum metabolites in rats. (**A**–**D**) OPLS-DA score scatter plot of serum metabolites (*n* = 7). (**E**–**H**) Volcano plot of differential metabolites (*n* = 7). The green dots indicate down-regulated metabolites, and the red dots indicate up-regulated metabolites (*p* < 0.05, FC > 1.5, FC < 0.67, and VIP > 1). (**I**–**L**) KEGG pathway analysis between two different groups.

Metabolomics data were used for data analysis through the HMDB database and the MetaboAnalyst website. First, OPLS-DA analysis was performed, which is a crucial step. Results showed that a good separation was observed at the PC1 level between the CON and MOD groups, the MOD and the treatment groups, suggesting substantial differences in metabolite expression between groups (Figure 5A–D). Differential metabolites between groups were screened according to *p* < 0.05, FC > 1.5, FC < 0.67, and VIP > 1, and volcano plots were drawn (Figure 5E–H). Serum untargeted metabolomics showed that 29 differential metabolites were present between the CON and MOD groups (Table S1), 17 differential metabolites between the MOD and POS groups (Table S2), 21 differential metabolites between the MOD and GP-H groups (Table S3) and 14 differential metabolites between the MOD and the GP-L (Table S4). Venn analysis of the screened differential metabolites showed that seven common differential metabolites were present among the groups, which were phosphate, mannitol, threonic acid, glyceric acid, lyxose, L-aspartic acid, and glutamine (Figure S2).

The protective effect of glutamine on the gut has been proven [43]. Glutamine supplementation can increase ZO-1 expression [44,45]. Glutamine is the primary source of energy for intestinal epithelial cells in the body and helps to maintain intestinal immune barrier function [46,47]. Additionally, it can manage the quantity of intestinal microbes and boost the use of amino acids within the intestines [48]. The primary regulation of glutamine release and its availability in the circulation is handled by key metabolic organs, such as the gut, liver, and skeletal muscles. In immune cells, glutamine is broken down into glutamate, aspartic acid, and alanine. This unique transformation pathway of glutamine is essential for the function of immune cells [49]. Another differential metabolite, L-aspartic acid, has been demonstrated to promote the aggravation of intestinal fibrosis in rats in vivo [50]. L-aspartic acid is a key metabolite of the action of Danggui Buxue Tang, which is clinically used for blood and qi deficiency and has a promoting effect on bone marrow hematopoietic function [51]. The elevated levels of L-aspartic acid indicated that the immune damage was being repaired [52]. The aforementioned previous studies could explain the down-regulation of the glutamine content and the up-regulation of the aspartic acid content in the serum of the administration group in this study.

#### 3.6.2. Metabolic Pathway Analysis

On the basis of the differential metabolites between groups, the enrichment of the metabolic pathways was performed, and 10 metabolic pathways with significant effects were screened out, involving carbohydrate metabolism, energy metabolism, and amino acid metabolism. Among them, amino acid metabolism pathways accounted for the majority, and the three most significant metabolic pathways were alanine, aspartate, and glutamate metabolism; arginine biosynthesis; and glyoxylate and dicarboxylate metabolism. The side effect of cyclophosphamide is bone marrow suppression, which makes it difficult to form blood, leading to anemia and immunodeficiency. The alanine, aspartate, and glutamate metabolism pathways are often associated with metabolic liver diseases [53]. This is consistent with the basic theory of traditional Chinese medicine that “the liver is the main reservoir of blood”. Arginine is a semi-essential amino acid that is a precursor to various molecules such as urea, polyamine, proline, and glutamic acid [54]. Tea and *Huangshui* polysaccharides benefit the body by altering the alanine, aspartate, and glutamate metabolism; arginine biosynthesis; and glyoxylate and dicarboxylate metabolic pathways [55,56]. These three metabolic pathways are often associated with immune-related diseases [57,58,59]. Overall, glutamate, glutamine, and arginine may be biomarkers for evaluating the efficacy of GPs.

### 3.7. Analysis of SCFAs Content in Serum and Faeces

**Figure 6:**
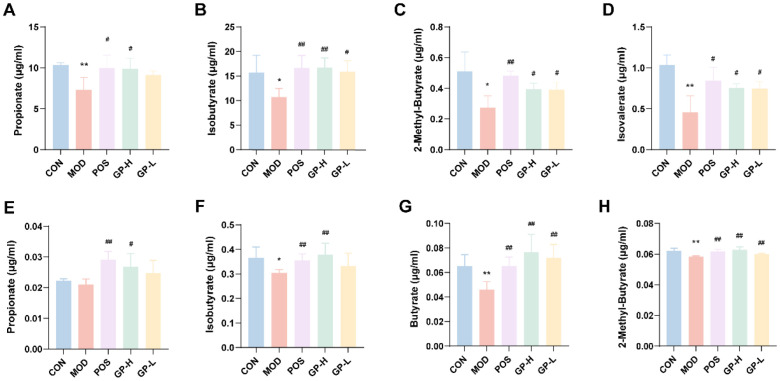
The concentration of SCFAs in rat fecal and serum samples in different groups. (**A**–**D**) SCFAs in fecal. (**E**–**H**) SCFAs in serum. * *p* < 0.05, ** *p* < 0.01 vs. the control group; ^#^ *p* < 0.05, ^##^ *p* < 0.01 vs. the model group.

SCFAs act as an essential bridge between diet, gut microbiota, metabolism, immunity, and cancer. The results of the determination of the content of SCFAs are shown in Figure 6. The contents of propionate, iso-butyrate, 2-methyl-butyrate, and isovalerate in feces were significantly increased after treatment with GPs (*p* < 0.05, Figure 6A–D). Correspondingly, GPs increased the levels of propionate, iso-butyrate, butyrate, and 2-methyl-butyrate in the serum of immunocompromised rats (*p* < 0.05, Figure 6E–H). Results showed that GPs alleviated immune damage and improved intestinal immune function by regulating the SCFAs content, especially propionate and butyrate. Propionate and butyrate are the major SCFAs, that contribute to the regulation of immunity, apoptosis, inflammation, and lipid metabolism [60]. Butyrate is involved in maintaining the integrity and function of the intestinal mucosal barrier through different mechanisms [61]. The main function of goblet cells is to secrete mucin, which is essential for the intrinsic barrier of the gut [62], and butyrate can directly induce polarized goblet cells, thereby maintaining the integrity of the intestinal barrier [63]. Butyric acid enhances intestinal barrier function by activating GPR43 [64]. Propionic acid is involved in the immunomodulation of various diseases. Propionic acid regulates Treg/Th17 balance by mediating IL-10 to alleviate multiple sclerosis [65]. Propionate may alleviate vascular calcification by improving intestinal barrier function and weakening the inflammatory response through gut microbiota remodeling [66]. The changes in the content of propionate and butyrate provided essential clues to the pharmacodynamic mechanism of GPs.

### 3.8. Correlation Analysis of Metabolites with Bacterial Species

**Figure 7:**
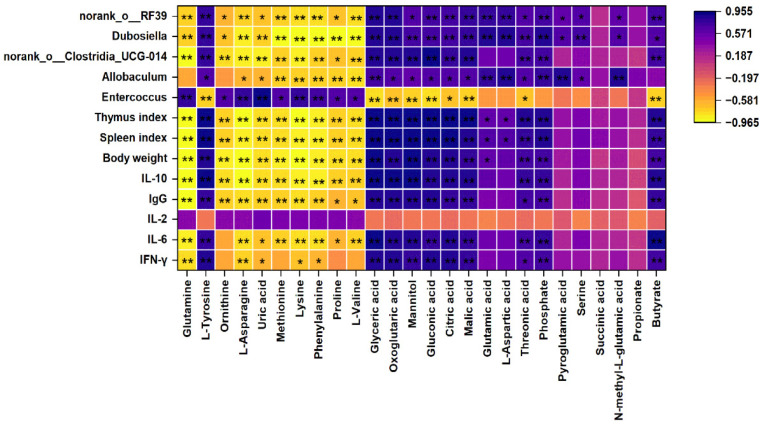
Correlation analysis between dominant gut microbiota with differential metabolites (CON vs. MOD). * *p* < 0.05, ** *p* < 0.01.

Correlation analysis of serum differential metabolites with body weight, spleen index, thymus index, cytokines, differential bacteria, and SCFAs was conducted (Figure 7). Glutamine, ornithine, L-asparagine, uric acid, methionine, lysine, phenylalanine, proline, and L-valine were significantly negatively correlated with physicochemical parameters, differential bacteria, and SCFAs. L-tyrosine, glyceric acid, and oxoglutaric acid, mannitol, gluconic acid, malic acid, threonic acid, and phosphate were significantly positively correlated with physicochemical parameters, differential bacteria, and SCFAs. Amino acid synthesis mainly occurs in the liver. Glutamic acid types are derived from α-ketoglutarate, including glutamic acid, glutamine, proline, and arginine [67]. Arginase mediates the hydrolysis of arginine to ornithine and urea [68]. Malic acid and aspartic acid are products of glutamine entering the TCA cycle for breakdown [69]. Glutamine, proline, ornithine, malic acid, aspartic acid, and glutamic acid are closely linked to the intestinal flora (Figure 7). Overall, the mechanism by which GPs regulate immune function may be contained in the gut–liver axis.

In particular, butyrate was negatively correlated with *Enterococcus* and positively correlated with *norank_o__Clostridia_UCG-014*, *Dubosiella,* and *norank_o__RF39*. On the basis of the in vitro culture and gene sequencing, butyric–acid-producing bacteria in the human intestine have been identified mainly from Lachnospiraceae, Ruminococcaceae, Clostridiaceae, Eubacteriaceae and Oscillospiraceae [70,71,72]. *Ruminococcus*, *Clostridium,* and *Faecalibacterium* contain specific species that produce butyrate [73]. A significant positive correlation is observed between butyrate and IL-10. Relevant studies have shown that butyrate inhibits IL-10 in gastric cancer tumor-associated macrophages [74]. The intestine, being the largest immune organ, is home to 70% of the immune cells of the body. The activation of immune cells by polysaccharides requires specific receptors. Cytokines, including interleukin, tumor necrosis factor, and interferon, play a crucial role in controlling inflammatory and immune reactions, influencing both innate and adaptive immunity [75]. Many anti-tumor mechanisms are attributed to immunomodulatory effects, and metabolic diseases such as obesity and diabetes are inseparable from intestinal microbiota disorders [55,76,77]. Butyrate exerts an immunomodulatory effect on neutrophils, thereby improving inflammatory bowel disease [78]. Similarly, butyrate acts on immune cells in allergic asthma [79].

The molecular weight, monosaccharide composition, linkage, and type of glycosidic bond of the polysaccharide will affect its activity [3]. The ability of polysaccharides to modulate intestinal microbiota varies because of their distinct structures [56]. Fermentation processes that degrade polysaccharides are advantageous for boosting their biological activity [80]. Overall, the breakdown of polysaccharides by the intestinal flora is conducive to improving the biological activity of polysaccharides.

Herein, the molecular weight, monosaccharide composition, functional groups, and microstructure of GPs were demonstrated. Subsequently, the therapeutic effect of GPs on immunocompromised rats was verified. Then, the mechanism by which GPs regulated immunity from the perspectives of gut microbiota and serum and fecal metabolomics was elucidated. In conclusion, GPs influence the immune system by altering the composition of gut microbiota and the levels of key metabolites. The possible metabolic pathways were alanine, aspartate, and glutamate metabolism; arginine biosynthesis; and glyoxylate and dicarboxylate metabolism, which provided research data for extending the clinical application of GPs.

## 4. Conclusions

Herein, the structural characteristics of GPs were initially elucidated, and their specific effects on the intestinal microbiota of immunocompromised rats were investigated. Several genera that interact with GPs were screened, which affirmed the prebiotic effects of GPs. These results lay the groundwork and basis for the subsequent screening of intestinal bacteria targeted by GPs and pave the way for GPs to become convenient and available functional foods. Employing both non-targeted and targeted metabolomics approaches, we examined the alterations in serum and fecal metabolites in immunocompromised rats after GP intervention. SCFAs are one of the more prominent compounds, in addition to many amino acids, which are also effectors of GPs that enrich the nutritional and functional characteristics of GPs. Building upon these findings, future research will focus on discovering the function and application of *Glycyrrhiza* polysaccharides in the food field. Due to their role in immune regulation, GPs have the potential to be developed into health foods and special dietary foods to enhance immunity, anti-fatigue, and restore energy.

## Figures and Tables

**Table 1:** Molecular weight distribution of GPs.

Rt (min)	lg M_W_	M_W_ (Da)
8.25	6.67	4.67 × 10^6^
8.78	6.34	2.19 × 10^6^
9.41	5.94	8.71 × 10^5^

## Data Availability

The original contributes presented in this study are included in the article/Supplementary Materials. Further inquires can be directed to the cooresponding author.

## Supplementary Materials

The following supporting information can be downloaded at: https://www.mdpi.com/article/10.3390/foods14050874/s1.

## References

[1] van Dinteren S., Meijerink J., Witkamp R., van Ieperen B., Vincken J.P., Araya-Cloutier C. (2022). Valorisation of liquorice (*Glycyrrhiza*) roots: Antimicrobial activity and cytotoxicity of prenylated (iso)flavonoids and chalcones from liquorice spent (*G. glabra*, *G. inflata*, and *G. uralensis*). Food Funct..

[2] Bakr A.F., Shao P., Farag M.A. (2022). Recent advances in glycyrrhizin metabolism, health benefits, clinical effects and drug delivery systems for efficacy improvement; a comprehensive review. Phytomedicine.

[3] Simayi Z., Rozi P., Yang X.J., Ababaikeri G., Maimaitituoheti W., Bao X.W., Ma S.J., Askar G., Yadikar N. (2021). Isolation, structural characterization, biological activity, and application of *Glycyrrhiza polysaccharides*: Systematic review. Int. J. Biol. Macromol..

[4] Aipire A., Yuan P.F., Aimaier A., Cai S.S., Mahabati M., Lu J., Ying T.L., Zhang B.H., Li J.Y. (2020). Preparation, Characterization, and Immuno-Enhancing Activity of Polysaccharides from *Glycyrrhiza uralensis*. Biomolecules.

[5] Wang Y.G., Wang X.J., Zhang K., Zhang X., Li S.W., Li Y.L., Fan W.G., Leng F.F., Yang M.J., Chen J.X. (2020). Extraction kinetics, thermodynamics, rheological properties and anti-BVDV activity of the hot water assisted extraction of *Glycyrrhiza* polysaccharide. Food Funct..

[6] Cheng A., Wan F., Jin Z., Wang J., Xu X. (2008). Nitrite oxide and inducible nitric oxide synthase were regulated by polysaccharides isolated from *Glycyrrhiza uralensis* Fisch. J. Ethnopharmacol..

[7] Aipire A., Mahabati M., Cai S.S., Wei X.X., Yu P.F., Aimaier A., Wang X.H., Li J.Y. (2020). The immunostimulatory activity of polysaccharides from *Glycyrrhiza uralensis*. PeerJ.

[8] Li J., Jia J., Teng Y., Wang X., Xia X., Song S., Zhu B., Xia X. (2025). Polysaccharides from Sea Cucumber (*Stichopus japonicus*) Synergize with Anti-PD1 Immunotherapy to Reduce MC-38 Tumor Burden in Mice Through Shaping the Gut Microbiome. Foods.

[9] Geng X., Tian W., Zhuang M., Shang H., Gong Z., Li J. (2024). Green Radish Polysaccharides Ameliorate Hyperlipidemia in High-Fat-Diet-Induced Mice via Short-Chain Fatty Acids Production and Gut Microbiota Regulation. Foods.

[10] Ayeka P.A., Bian Y.H., Githaiga P.M., Zhao Y. (2017). The immunomodulatory activities of licorice polysaccharides (*Glycyrrhiza uralensis* Fisch.) in CT 26 tumor-bearing mice. BMC Complement. Altern. Med..

[11] Cheng Z.R., Zheng Q., Duan Y.Q., Cai M.H., Zhang H.H. (2024). Effect of subcritical water temperature on the structure, antioxidant activity and immune activity of polysaccharides from *Glycyrrhiza inflata* Batalin. Int. J. Biol. Macromol..

[12] Zhang C.H., Yu Y., Liang Y.Z., Chen X.Q. (2015). Purification, partial characterization and antioxidant activity of polysaccharides from *Glycyrrhiza uralensis*. Int. J. Biol. Macromol..

[13] Cockburn D.W., Koropatkin N.M. (2016). Polysaccharide Degradation by the Intestinal Microbiota and Its Influence on Human Health and Disease. J. Mol. Biol..

[14] Wu L., Gao Y., Su Y., Li J., Ren W.C., Wang Q.H., Kuang H.X. (2022). Probiotics with anti-type 2 diabetes mellitus properties: Targets of polysaccharides from traditional Chinese medicine. Chin. J. Nat. Medicines..

[15] Wei X.X., Li N., Wu X.Y., Cao G.D., Qiao H.P., Wang J., Hao R.R. (2023). The preventive effect of *Glycyrrhiza* polysaccharide on lipopolysaccharide-induced acute colitis in mice by modulating gut microbial communities. Int. J. Biol. Macromol..

[16] Song W.D., Wang Y.Y., Li G.C., Xue S.N., Zhang G.L., Dang Y.Y., Wang H.B. (2023). Modulating the gut microbiota is involved in the effect of low-molecular-weight *Glycyrrhiza* polysaccharide on immune function. Gut Microbes.

[17] Zhang D., Liu J., Cheng H., Wang H., Tan Y., Feng W., Peng C. (2022). Interactions between polysaccharides and gut microbiota: A metabolomic and microbial review. Food Res. Int..

[18] Wu Y., Wu C., Che Y., Zhang T., Dai C., Nguyen A.D., Duan K., Huang Y., Li N., Zhou H. (2022). Effects of *Glycyrrhiza* Polysaccharides on Chickens’ Intestinal Health and Homeostasis. Front. Vet. Sci..

[19] Qiao Y., Guo Y., Zhang W., Guo W., Oleksandr K., Bozhko N., Wang Z., Liu C. (2022). Effects of Compound Polysaccharides Derived from Astragalus and *Glycyrrhiza* on Growth Performance, Meat Quality and Antioxidant Function of Broilers Based on Serum Metabolomics and Cecal Microbiota. Antioxidants.

[20] Meng M., Sun Y., Qi Y.L., Xu J., Sun J.G., Bai Y.H., Han L.R., Han R., Hou L.H., Sun H.Q. (2023). Structural characterization and induction of tumor cell apoptosis of polysaccharide from purple sweet potato (*Ipomoea batatas* (L.) Lam). Int. J. Biol. Macromol..

[21] Sun S.J., Deng P., Peng C.E., Ji H.Y., Mao L.F., Peng L.Z. (2022). Extraction, Structure and Immunoregulatory Activity of Low Molecular Weight Polysaccharide from *Dendrodium officinale*. Polymers.

[22] Yang Y.R., Zhang Y.W., Song J.P., Li Y.Q., Zhou L.Y., Xu H.T., Wu K.Z., Gao J., Zhao M.M., Zheng Y. (2023). Bergamot polysaccharides relieve DSS-induced ulcerative colitis via regulating the gut microbiota and metabolites. Int. J. Biol. Macromol..

[23] Wang D.P., Dong Y., Xie Y., Xiao Y.X., Ke C., Shi K., Zhou Z.S., Tu J.Y., Qu L.H., Liu Y.J. (2023). *Atractylodes lancea* Rhizome Polysaccharide Alleviates Immunosuppression and Intestinal Mucosal Injury in Mice Treated with Cyclophosphamide. J. Agric. Food Chem..

[24] Huang S.W., Ye Q.J., Wang A.J., Chen Y. (2024). *Paeoniae* Decoction restores intestinal barrier dysfunction by promoting the interaction between ILC3 and gut flora. Phytomedicine.

[25] Wu H., Chen Q.Y., Wang W.Z., Chu S., Liu X.X., Liu Y.J., Tan C., Zhu F., Deng S.J., Dong Y.L. (2021). Compound sophorae decoction enhances intestinal barrier function of dextran sodium sulfate induced colitis via regulating notch signaling pathway in mice. Biomed. Pharmacother..

[26] Zhang W., Ding M.L., Feng Y.R., Cai S.H., Luo Z.C., Shan J.J., Di L.Q. (2024). Modulation of cellular metabolism and alleviation of bacterial dysbiosis by *Aconiti lateralis radix praeparata* in non-small cell lung cancer treatment. Phytomedicine.

[27] Shan J.J., Peng L.X., Qian W.J., Xie T., Kang A., Gao B., Di L.Q. (2018). Integrated Serum and Fecal Metabolomics Study of Collagen-Induced Arthritis Rats and the Therapeutic Effects of the Zushima Tablet. Front. Pharmacol..

[28] Furuse M., Hirase T., Itoh M., Nagafuchi A., Yonemura S., Tsukita S. (1993). Occludin: A Novel Integral Membrane Protein Localizing at Tight Junctions. J. Cell Biol..

[29] Kuo W.T., Zuo L., Odenwald M.A., Madha S., Singh G., Gurniak C.B., Abraham C., Turner J.R. (2021). The Tight Junction Protein ZO-1 Is Dispensable for Barrier Function but Critical for Effective Mucosal Repair. Gastroenterology.

[30] Buckley A., Turner J.R. (2018). Cell Biology of Tight Junction Barrier Regulation and Mucosal Disease. Cold Spring Herb. Perspect. Biol..

[31] Al-Sadi R., Khatib K., Guo S.H., Ye D.M., Youssef M., Ma T. (2011). Occludin regulates macromolecule flux across the intestinal epithelial tight junction barrier. Am. J. Physiol. Gastrointest. Liver Physiol..

[32] Stojanov S., Berlec A., Strukelj B. (2020). The Influence of Probiotics on the Firmicutes/Bacteroidetes Ratio in the Treatment of Obesity and Inflammatory Bowel disease. Microorganisms.

[33] Gong H., Gan X.A., Qin B.Y., Chen J., Zhao Y.L., Qiu B.Y., Chen W.H., Yu Y., Shi S.S., Li T.Z. (2024). Structural characteristics of steamed *Polygonatum cyrtonema* polysaccharide and its bioactivity on colitis via improving the intestinal barrier and modifying the gut microbiota. Carbohydr. Polym..

[34] Xie N.N., Wu C.Y., Ge Q., Zhou J., Long F., Mao Q., Li S.L., Shen H. (2023). Structure-specific antitumor effects and potential gut microbiota-involved mechanisms of ginseng polysaccharides on B16F10 melanoma-bearing mice. Food Funct..

[35] Schiopu P., Toc D.A., Colosi I.A., Costache C., Ruospo G., Berar G., Galbau S.G., Ghilea A.C., Botan A., Pana A.G. (2023). An Overview of the Factors Involved in Biofilm Production by the Enterococcus Genus. Int. J. Mol. Sci..

[36] Sangiorgio G., Calvo M., Migliorisi G., Campanile F., Stefani S. (2024). The Impact of Enterococcus spp. in the Immunocompromised Host: A Comprehensive Review. Pathogens.

[37] Ye Y., Liu J.H., Zheng D., Zeng X.F., Zhou Z.X., Han L.T., Huang P., Zhang F.Y., Wang W.S., Cheng X. (2023). Serum Metabolomics Combined With 16S rRNA Gene Sequencing to Analyze the Changes of Intestinal Flora in Rats With MI and the Intervention Effect of Fuling-Guizhi. Nat. Prod. Commun..

[38] Gu W.C., Zhang L.K., Han T., Huang H.L., Chen J. (2022). Dynamic Changes in Gut Microbiome of Ulcerative Colitis: Initial Study from Animal Model. J. Inflamm. Res..

[39] Xiao X.Y., Guo Z.J., Li X.M., Chen P., Li Y., Zhang J.B., Mao C.Q., Ji D., Su L.L., Gao B. (2023). Effects of wine processed Polygonatum polysaccharides on immunomodulatory effects and intestinal microecology in mice. Qual. Assur. Saf. Crops Foods.

[40] Hu Q., Wu C.Y., Yu J.T., Luo J.M., Peng X.C. (2022). Angelica sinensis polysaccharide improves rheumatoid arthritis by modifying the expression of intestinal Cldn5, Slit3 and Rgs18 through gut microbiota. Int. J. Biol. Macromol..

[41] Zhang Y.N., Tu S.Y., Ji X.W., Wu J.N., Meng J.X., Gao J.S., Shao X., Shi S., Wang G., Qiu J.J. (2024). Dubosiella newyorkensis modulates immune tolerance in colitis via the L-lysine-activated AhR-IDO1-Kyn pathway. Nat. Commun..

[42] Li Z.W., Sang R.X., Feng G.L., Feng Y.X., Zhang R., Yan X.B. (2024). Microbiological and metabolic pathways analysing the mechanisms of alfalfa polysaccharide and sulfated alfalfa polysaccharide in alleviating obesity. Int. J. Biol. Macromol..

[43] Beutheu S., Ghouzali I., Galas L., Déchelotte P., Coëffier M. (2013). Glutamine and arginine improve permeability and tight junction protein expression in methotrexate-treated Caco-2 cells. Clin. Nutr..

[44] Wu Q.J., Liu N., Wu X.H., Wang G.Y., Lin L. (2018). Glutamine alleviates heat stress-induced impairment of intestinal morphology, intestinal inflammatory response, and barrier integrity in broilers. Poult. Sci..

[45] Zhang J., Wu G.C., Shan A.S., Han Y., Jin Y.C., Fang H.T., Zhao Y., Shen J.L., Zhou C.H., Li C.J. (2017). Dietary glutamine supplementation enhances expression of ZO-1 and occludin and promotes intestinal development in Min piglets. Acta Agric. Scand. Sect. A Anim. Sci..

[46] Wang B., Wu G.Y., Zhou Z.G., Dai Z.L., Sun Y.L., Ji Y., Li W., Wang W.W., Liu C., Han F. (2015). Glutamine and intestinal barrier function. Amino Acids.

[47] Kim M.H., Kim H. (2017). The Roles of Glutamine in the Intestine and Its Implication in Intestinal Diseases. Int. J. Mol. Sci..

[48] Dai Z.L., Li X.L., Xi P.B., Zhang J., Wu G.Y., Zhu W.Y. (2013). L-Glutamine regulates amino acid utilization by intestinal bacteria. Amino Acids.

[49] Cruzat V., Rogero M.M., Keane K.N., Curi R., Newsholme P. (2018). Glutamine: Metabolism and Immune Function, Supplementation and Clinical Translation. Nutrients.

[50] Li X.H., Hu S.X., Shen X.D., Zhang R.A., Liu C.G., Xiao L., Lin J.J., Huang L., He W.T., Wang X.Y. (2024). Multiomics reveals microbial metabolites as key actors in intestinal fibrosis in Crohn’s disease. EMBO Mol. Med..

[51] Ji P., Li C.C., Wei Y.M., Hua Y.L., Yao W.L., Wu F.L., Zhang X.S., Yuan Z.W., Zhao N.S., Zhang Y.H. (2022). A new method providing complementary explanation of the blood-enriching function and mechanism of unprocessed *Angelica sinensis* and its four kinds of processed products based on tissue-integrated metabolomics and confirmatory analysis. Biomed. Chromatogr..

[52] Wang X.H., Zhang T., Li W.L., Zhang M.G., Zhao L.W., Wang N.X., Zhang X.W., Zhang B.B. (2024). Dietary supplementation with *Macleaya cordata* extract alleviates intestinal injury in broiler chickens challenged with lipopolysaccharide by regulating gut microbiota and plasma metabolites. Front. Immunol..

[53] Sookoian S., Pirola C.J. (2012). Alanine and aspartate aminotransferase and glutamine-cycling pathway: Their roles in pathogenesis of metabolic syndrome. World J. Gastroenterol..

[54] Zhang Y.M., Higgins C.B., Tica S., Adams J.A., Sun J.M., Kelly S.C., Zong X.Y., Dietzen D.J., Pietka T., Ballentine S.J. (2024). Hierarchical tricarboxylic acid cycle regulation by hepatocyte arginase 2 links the urea cycle to oxidative metabolism. Cell Metab..

[55] Li H.S., Fang Q.Y., Nie Q.X., Hu J.L., Yang C., Huang T., Li H., Nie S.P. (2020). Hypoglycemic and Hypolipidemic Mechanism of Tea Polysaccharides on Type 2 Diabetic Rats via Gut Microbiota and Metabolism Alteration. J. Agric. Food Chem..

[56] Li M., Su J., Wu J.H., Zhao D., Huang M.Q., Lu Y.P., Zheng J., Zheng F.P., Sun B.G., Liang H.Y. (2024). The Regulatory Effect of Huangshui Polysaccharides on Intestinal Microbiota and Metabolites during In Vitro Fermentation. J. Agric. Food Chem..

[57] Yuan Q.H., Deng D.W., Pan C., Ren J., Wei T.F., Wu Z.M., Zhang B., Li S., Yin P.Y., Shang D. (2022). Integration of transcriptomics, proteomics, and metabolomics data to reveal HER2-associated metabolic heterogeneity in gastric cancer with response to immunotherapy and neoadjuvant chemotherapy. Front. Immunol..

[58] Zhao Y., Ma C.C., Cai R.Z., Xin L.J., Li Y.S., Ke L.X., Ye W., Ouyang T., Liang J.H., Wu R.H. (2024). NMR and MS reveal characteristic metabolome atlas and optimize esophageal squamous cell carcinoma early detection. Nat. Commun..

[59] Cong S., Wang L.R., Meng Y., Cai X.L., Zhang C.X., Gu Y.Q., Ma X.M., Luo L. (2023). Saussurea involucrata oral liquid regulates gut microbiota and serum metabolism during alleviation of collagen-induced arthritis in rats. Phytother. Res..

[60] Zhang D., Jian Y.P., Zhang Y.N., LI Y., Gu L.T., Sun H.H., Liu M., Zhou H.L., Wang Y.S., Xu Z.X. (2023). Short-chain fatty acids in diseases. Cell Commun. Signal..

[61] Mann E.R., Lam Y.K., Uhlig H.H. (2024). Short-chain fatty acids: Linking diet, the microbiome and immunity. Nat. Rev. Immunol..

[62] Zhang J., Yu Q.Z., Jiang D.L., Yu K., Yu W.W., Chi Z.X., Chen S., Li M.B., Yang D.H., Wang Z. (2022). Epithelial Gasdermin D shapes the host-microbial interface by driving mucus layer formation. Sci. Immunol..

[63] Gaudier E., Jarry A., Blottière H.M., de Coppet P., Buisine M.P., Aubert J.P., Laboisse C., Cherbut C., Hoebler C. (2004). Butyrate specifically modulates MUC gene expression in intestinal epithelial goblet cells deprived of glucose. Am. J. Physiol. -Gastrointest. Liver Physiol..

[64] D’Souza W.N., Douangpanya J., Mu S.R., Jaeckel P., Zhang M., Maxwell J.R., Rottman J.B., Labitzke K., Willee A., Beckmann H. (2017). Differing roles for short chain fatty acids and GPR43 agonism in the regulation of intestinal barrier function and immune responses. PLoS ONE.

[65] Duscha A., Gisevius B., Hirschberg S., Yissachar N., Stangl G.I., Dawin E., Bader V., Haase S., Kaisler J., David C. (2020). Propionic Acid Shapes the Multiple Sclerosis Disease Course by an Immunomodulatory Mechanism. Cell.

[66] Yan J.L., Pan Y.B., Shao W.M., Wang C.P., Wang R.N., He Y.Q., Zhang M., Wang Y.S., Li T.Z.M., Wang Z.F. (2022). Beneficial effect of the short-chain fatty acid propionate on vascular calcification through intestinal microbiota remodelling. Microbiome.

[67] Altman B.J., Stine Z.E., Dang C.V. (2016). From Krebs to clinic: Glutamine metabolism to cancer therapy. Nat. Rev. Cancer.

[68] Morris S.M. (2007). Arginine metabolism: Boundaries of our knowledge. J. Nutr..

[69] Guo J.W., Yu J., Peng F., Li J.Z., Tan Z.R., Chen Y., Rao T., Wang Y.C., Peng J.B., Zhou H.H. (2021). In vitro and in vivo analysis of metabolites involved in the TCA cycle and glutamine metabolism associated with cisplatin resistance in human lung cancer. Expert Rev. Proteom..

[70] Fu X.D., Liu Z.M., Zhu C.L., Mou H.J., Kong Q. (2019). Nondigestible carbohydrates, butyrate, and butyrate-producing bacteria. Crit. Rev. Food Sci. Nutr..

[71] Martin-Gallausiaux C., Marinelli L., Blottière H.M., Larraufie P., Lapaque N. (2021). SCFA: Mechanisms and functional importance in the gut. Proc. Nutr. Soc..

[72] Louis P., Flint H.J. (2017). Formation of propionate and butyrate by the human colonic microbiota. Environ. Microbiol..

[73] Stoeva M.K., Garcia-So J., Justice N., Myers J., Tyagi S., Nemchek M., McMurdie P.J., Kolterman O., Eid J. (2021). Butyrate-producing human gut symbiont, Clostridium butyricum, and its role in health and disease. Gut Microbes.

[74] Lee S.Y., Jhun J., Woo J.S., Lee K.H., Hwang S.H., Moon J., Park G., Choi S.S., Kim S.J., Jung Y.J. (2024). Gut microbiome-derived butyrate inhibits the immunosuppressive factors PD-L1 and IL-10 in tumor-associated macrophages in gastric cancer. Gut Microbes.

[75] Yin M., Zhang Y., Li H. (2019). Advances in Research on Immunoregulation of Macrophages by Plant Polysaccharides. Front. Immunol..

[76] Zhu Y.Z., Wang D., Zhou S.B., Zhou T. (2024). Hypoglycemic Effects of *Gynura divaricata* (L.) DC Polysaccharide and Action Mechanisms via Modulation of Gut Microbiota in Diabetic Mice. J. Agric. Food Chem..

[77] Ma Y., Xie H., Xu N., Li M.N., Wang L., Ge H.F., Xie Z.W., Li D.X., Wang H.Y. (2024). Large Yellow Tea Polysaccharide Alleviates HFD-Induced Intestinal Homeostasis Dysbiosis via Modulating Gut Barrier Integrity, Immune Responses, and the Gut Microbiome. J. Agric. Food Chem..

[78] Li G.F., Lin J., Zhang C., Gao H., Lu H.Y., Gao X., Zhu R.X., Li Z.T., Li M.S., Liu Z.J. (2021). Microbiota metabolite butyrate constrains neutrophil functions and ameliorates mucosal inflammation in inflammatory bowel disease. Gut Microbes.

[79] Yip W., Hughes M.R., Li Y.C., Cait A., Hirst M., Mohn W.W., McNagny K.M. (2021). Butyrate Shapes Immune Cell Fate and Function in Allergic Asthma. Front. Immunol..

[80] Ma J.J., Wu W.Y., Liao J., Liu L., Wang Q., Xiao G.S., Liu H.F. (2024). Preparation of Dendrobium officinale Polysaccharide by Lactic Acid Bacterium Fermentation and Its Protective Mechanism against Alcoholic Liver Damage in Mice. J. Agric. Food Chem..

