# Supplementary material for: Immunomodulation of Glycyrrhiza Polysaccharides In Vivo Based on Microbiome and Metabolomics Approaches
Source: Foods. 2025 Mar 4;14(5):874. doi: 10.3390/foods14050874 (PMC11898905; doi:10.3390/foods14050874)

Supplementary Information

1. Determination of polysaccharide content.

The phenol–sulfuric acid method was used to measure the total sugar content of GP. To prepare the reference solution, 25 mg of glucose reference substance was precisely weighed and placed in a 250 mL volumetric flask, then diluted to the mark with distilled water. A standard curve was created by placing 0.2, 0.4, 0.6, 0.8, and 1.0 mL of a 0.1 mg/mL glucose solution into a dry stoppered test tube, then adding water to reach a total volume of 2 mL. Precisely 1 mL of a 5% phenol solution was added and mixed thoroughly, followed by the rapid and accurate addition of 5mL of concentrated sulfuric acid, mixed again, allowed to sit for 10 minutes, and then maintained in a 40 °C water bath for 15 minutes. Once removed, it was rapidly brought down to room temperature, and its absorbance was checked using a UV spectrophotometer at 490 nm, using the corresponding reagent as a blank. The standard curve was plotted with absorbance on the y-axis and glucose mass concentration on the x-axis.

To prepare the test solution, 10 mg of GP was precisely weighed, placed in a 100 mL volumetric flask, diluted to the mark with distilled water, and used as the test solution. For the test solution determination, 0.4 mL was carefully absorbed and placed in a dry, sealed test tube. Water was added to reach a total of 2 mL, and the absorbance was measured at 0.262 according to the specified conditions.

2. Determination of protein content.

Preparation of the protein standard solution: An accurately weighed 25 mg sample of bovine serum protein was dissolved in distilled water, and the solution was adjusted to a final volume of 250 mL.

Preparation of Coomassie Brilliant Blue G-250 Solution: A total of 100 mg of Coomassie Brilliant

Blue G-250 was dissolved in 50 mL of 90% ethanol. Subsequently, 100 mL of 85% phosphoric acid was added, and the solution was brought to a final volume of 1000 mL with distilled water. Construction of the standard curve: Aliquots of 0.2, 0.4, 0.6, 0.8, and 1.0 mL of the protein standard solution were transferred into dry centrifuge tubes. Each aliquot was diluted to 1 mL with distilled water, followed by the addition of 5 mL of Coomassie Brilliant Blue G-250 solution. The mixtures were thoroughly mixed and allowed to stand for 2 minutes. Absorbance measurements were conducted using a UV spectrophotometer at a wavelength of 595 nm, with the corresponding reagent serving as the blank. The absorbance values were plotted on the ordinate against the protein mass on the abscissa to generate the standard curve.

Preparation of test solution: An accurately weighed 8.5 mg sample of GP was placed in a 5 mL volumetric flask, diluted to the mark with distilled water, and used as the test solution. Determination of the test solution: A 0.1 mL of the test solution was precisely measured and transferred into a dry centrifuge tube. Subsequently, water was added to achieve a total volume of 1 mL, and the absorbance was recorded at 0.239, in accordance with the conditions established for the standard curve.

3. Determination of uronic acid content.

Reagent Preparation: A quantity of 0.15 g of m-hydroxybiphenyl was accurately weighed and dissolved in a sodium hydroxide solution at a concentration of 5 mg/mL, with the final volume adjusted to 100 mL. Additionally, 0.478 g of sodium tetraborate was precisely weighed and dissolved in 100 mL of concentrated sulfuric acid for subsequent use. Furthermore, 25 mg of galacturonic acid, previously dried to a constant weight, was dissolved in water, and the volume was adjusted to 25 mL to prepare a standard solution with a concentration of 1 mg/mL. Preparation of

standard curve: Aliquots of 0.1, 0.2, 0.3, 0.4, 0.5, and 0.6 mL of the 1 mg/mL galactose standard solution were transferred into separate 10 mL volumetric flasks, and the volumes were adjusted with water. Subsequently, 1.0 mL of each prepared solution was placed into a 20 mL stoppered test tube, which was then immersed in an ice-water bath. Following this, 6 mL of the sodium tetraborate/sulfuric acid solution was added to each tube. After thorough mixing, the tubes were heated in a boiling water bath for 5 minutes. Upon cooling, 100 μL of a 1.5 mg/mL meta-hydroxybiphenyl solution was added to each tube. The mixture was thoroughly combined and subjected to shaking for 5 minutes, followed by ultrasonication to eliminate air bubbles. The absorbance was measured using a UV spectrophotometer at a wavelength of 525 nm, employing the corresponding reagent as a blank. The absorbance values were plotted on the ordinate, while the protein mass was plotted on the abscissa to construct the standard curve.

Preparation of the test solution involved accurately weighing 8.5 mg of GP and placing it in a 5 mL volumetric flask, which was then filled to the mark with distilled water to serve as the test solution. For the determination of the test solution, 0.1 mL was precisely aspirated and transferred into a sealed test tube, followed by the addition of water to reach a total volume of 2 mL. The absorbance was then measured, yielding a value of 0.387, in accordance with the conditions established for the standard curve.

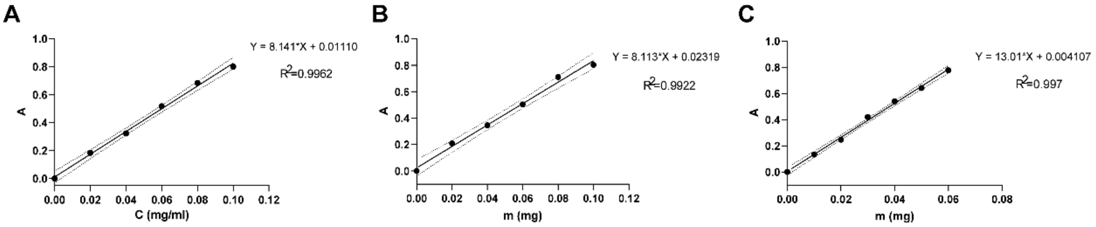

Figure S1. The basic properties of GP. (A) Standard curve of glucose content. (B) Standard curve for protein content. (C) Standard curve of uronic acid content.

Table S1. Differential metabolites between MOD and CON.

Serial number	Up	Down
1	Glutamine	Glyceric acid
2	Tyrosine	Alpha-ketoglutarate
3	Ornithine	Mannitol
4	L-Asparagine	Gluconic acid
5	Uric acid	Citric acid
6	Isopropyl-beta-D-	L-Tyrosine
7	Methyl-phosphate	Malic acid
8	Methionine	Glutamic acid
9	Lysine	L-Aspartic acid
10	Phenylalanine	Lyxose
11	Proline	Threonic acid
12	L-Valine	Phosphate
13		Oxo-proline
14		Serine
15		Succinic acid
16		1,2,4-benzenetriol
17		N-methyl-L-glutamic acid

Table S2. Differential metabolites between POS and MOD.

Serial number	Up	Down
1	Mannitol	Glutamine
2	Glyceric acid	
3	Citric acid	
4	Phosphate	
5	Gluconic acid	
6	Glutamate	
7	Alpha-ketoglutarate	
8	Threonic acid	
9	O-phosphorylethanolamine	
10	Threo-beta-hydroxy-aspartate	
11	Glucose-6-phosphate	
12	Malic acid	
13	Lyxose	
14	L-Tyrosine	
15	Glutamic acid	
16	L-Aspartic acid	
17	N-methyl-L-glutamic Acid	

Table S3. Differential metabolites between GP-H and MOD.

Serial number	Up	Down
1	Mannitol	2-Hydroxybutanoic acid
2	Phosphate	Isopropyl-beta-D-
3	Threo-beta-hydroxy-aspartate	Glutamine
4	Glyceric acid	Ornithine
5	Citric acid	Methyl-phosphate
6	1,2,4-benzenetriol	
7	O-Phosphoethanolamine	
8	Lyxose	
9	Threonic acid	
10	5-Aminovaleric acid	
11	Glutamic acid	
12	L-Aspartic acid	
13	L-Tyrosine	
14	Succinic acid	
15	m-Cresol	
16	N-methyl-L-glutamic Acid	

Table S4. Differential metabolites between GP-L and MOD.

Serial number	Up	Down
1	Phosphate	Glutamine
2	Mannitol	Ornithine
3	Threonic acid	Isopropyl-beta-D-
4	Glyceric acid	Putrescine
5	Lyxose	
6	Glucose-6-phosphate	
7	Malic acid	
8	Succinic acid	
9	L-Aspartic acid	
10	Gluconic acid	

Figure S2. Shared differential metabolites.

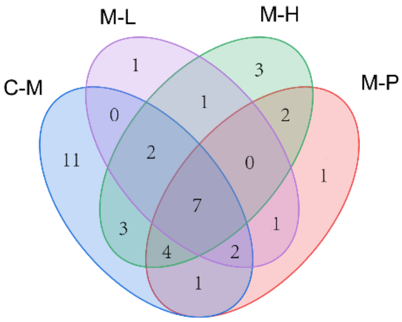

Supplement: Supplementary file 1 [file foods-14-00874-s001.zip › foods-3491234-supplementary.pdf]
